# Case Report: Novel Coronavirus—A Potential Cause of Acute Pancreatitis?

**DOI:** 10.4269/ajtmh.20-0568

**Published:** 2020-07-08

**Authors:** Syed Muhammad Mashhood Ali Bokhari, Fatima Mahmood

**Affiliations:** Nishtar Medical College and Hospital, Multan, Pakistan

## Abstract

A 32-year-old medical practitioner presented to the emergency department with complaints of severe abdominal pain, fever, and vomiting, 1 week after the diagnosis of COVID-19. The patient did not report any comorbid conditions, alcohol usage, or gallstone disease. Laboratory and radiological investigations revealed the diagnosis of acute pancreatitis. He underwent conservative management and was discharged after 3 days of hospital admission. This case highlights a possible association between COVID-19 and acute pancreatitis, and the need for clinicians to carefully evaluate patients presenting with gastrointestinal complaints during the current pandemic.

## CASE DESCRIPTION

A 32-year-old male patient presented to the outpatient clinic at Nishtar Hospital, Multan, Pakistan, with a history of relapsing fever, sore throat, productive cough, myalgia, and diarrhea for 1 week. The patient was a medical physician currently working in the medicine ward. He did not report any prior comorbidities or high-risk travel history.

Considering the exposure history, a reverse transcriptase–PCR for COVID-19 was obtained. Initially, the patient’s temperature was 38°C, the blood pressure 110/80 mmHg, the pulse rate 110 beats per minute, the respiratory rate 16 breaths per minutes, and oxygen saturation 99–100% while breathing in ambient air. He was managed with simple supportive measures.

After 21 hours, his tests for COVID-19 were reported positive. The patient chose to self-isolate at home until his symptoms subsided and his results came negative. He was advised to report to the emergency department in case of severe dyspnea, chest pain, cyanosis, or altered mental status.

A week after the diagnosis, the patient reported to the emergency department with severe mid-epigastric pain radiating to the back accompanied by intermittent high fevers, chills, and non-biliary vomiting. He was noted to have a white cell count of 12 × 10^9^/L and glucose levels of 192 mg/dL. Liver function tests revealed aspartate aminotransferase of 29 IU/L and alanine aminotransferase of 27 IU/L. Kidney functions were normal, serum triglycerides were 150 mg/dL, and serum calcium was 8.9 mg/dL. Serum amylase was 672 IU/L and serum lipase 721 IU/L (Table 1). The patient reported no history of use of alcohol.

**Table 1:** Clinical laboratory results

Variable	Reference range	Day 1	Day 2
C-reactive protein (mg/dL)	0–0.5	1.58	–
Lipase (U/L)	6–51	721	380
Amylase (U/L)	28–100	672	–
Glucose (mg/dL)	80–160	192	153
Blood urea nitrogen (mg/dL)	6–20	11	–
Creatinine (mg/dL)	0.9–1.3	1.3	1.1
Sodium (mmol/L)	136–145	136	140
Potassium (mmol/L)	3.5–5.1	3.7	4.2
Chloride (mmol/L)	98–107	101	107
Calcium (mg/dL)	8.6–10.2	8.9	–
Bicarbonate (mmol/L)	20–31	20.4	26.4
Lactate dehydrogenase (U/L)	120–246	212	–
Aspartate aminotransferase (U/L)	10–40	29	–
Alanine aminotransferase (U/L)	10–49	27	–
Alkaline phosphatase (U/L)	46–116	82	–
Total bilirubin (mg/dL)	0.1–1.2	1.0	–
Total cholesterol			
Triglyceride		150	–
Hemoglobin (g/dL)	13.7–16.3	14.2	–
Hematocrit (%)	41.9–48.7	42.8	–
White blood count (×10^9^/L)	4.0–10.0	12	11.2
Platelets (×10^9^/L)	150–400	247	–

**Figure 1.**
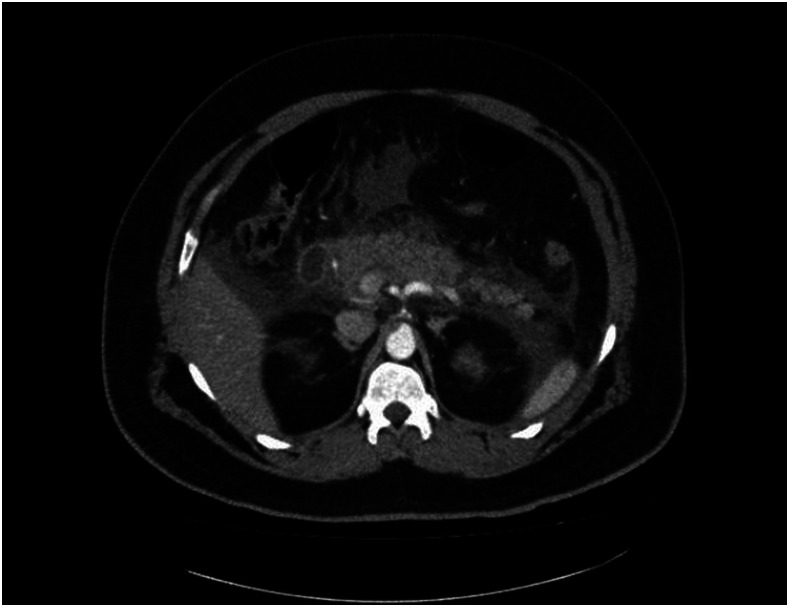
CT scan showing a swollen pancreas with inflammatory changes in surrounding areas.

Ultrasound in the emergency ward showed no signs of inflammation of the gall bladder or cholelithiasis. Computed axial tomography scan of the abdomen revealed a bulky and swollen pancreas with significant peripancreatic inflammatory changes and fluid collection along the gastrosplenic ligament. Otherwise, the pancreas was non-enhancing, with no evidence of low-attenuation areas in the pancreatic parenchyma to suggest necrosis (Figure 1).

Considering a diagnosis of acute pancreatitis, the patient was kept nothing per oral and on intravenous fluids, analgesics, antibiotics, and antiemetics. The patient’s intake and output were monitored, and he was gradually mobilized from bed. Alongside, incentive spirometry and chest physiotherapy were initiated.

Over the next 3 days, his lipase was constantly monitored and showed a downward trend, 380, 198, and 56 IU/L. On day 3, his symptoms improved, and he was started on clear fluids and soft diet through oral route, which was successfully tolerated. The patient was discharged home without any sequelae.

## DISCUSSION

In early December 2019, Wuhan City, China, experienced a sudden outbreak of pneumonia. Investigation led to isolation of a novel coronavirus as the cause of outbreak. The WHO named it as COVID-19 on February 11, 2020. This virulent pathogen rapidly spread in China and other regions in the world. Till date, the virus continues to cause a devastating pandemic worldwide, threatening global health and affecting approximately 4,000,000 people. It causes great threats to the growth of economy and society.^1,2^

Respiratory symptoms such as cough, shortness of breath, and sore throat appear to be the pathognomonic signs in a patient infected with the coronavirus; however, the literature regarding its extrapulmonary symptoms are still evolving. With the increase in the number of cases and accumulation of data on possible signs and symptoms, it appears that gastrointestinal symptoms are fairly common in COVID-19 patients. A comprehensive study conducted in Hubei, China, evaluated 204 patients who tested positive for COVID-19, of whom 50.5% reported some gastrointestinal disturbance such as diarrhea, anorexia, vomiting, or abdominal pain. A rare group of patients presented with gastrointestinal symptoms only without any respiratory symptoms.^3^

In our case, the diagnosis of pancreatitis in this patient appears to be idiopathic in the absence of comorbid conditions, cholelithiasis, alcohol usage, trauma, or recent invasive procedures such as endoscopic retrograde cholangiopancreatography. However, around 10% of cases are due to miscellaneous factors such as viral, bacterial, or parasitic infections. Notably, Coxsackievirus, herpes simplex virus, mumps, human immunodeficiency virus, and *Mycoplasma*, among several others, are responsible for causing infectious pancreatitis.^4^ Similar cases have been reported recently from Newport (United Kingdom) and Denmark, where multiple patients with COVID-19 disease presented with complaints of acute pancreatitis.^5,6^ A study in Wuhan city showed the prevalence of pancreatic injury in nine of 52 patients admitted in a hospital. It suggested that the pancreatic injury could be due to heavy expression of angiotensin converting enzyme or harmful systemic immune response induced by COVID-19 infection.^7^ Based on this case and the aforementioned studies, we recommend further studies to be conducted to evaluate any possible association between COVID-19 and acute pancreatitis.

## CONCLUSION

This is a case describing the incidence of pancreatitis in a COVID-19–positive patient. We encourage medical practitioners to carefully evaluate gastrointestinal symptoms and measure serum amylase and lipase levels in patients presenting with abdominal pain and COVID-19.
